# Multidisciplinary Identification of the Controversial Freedom Fighter Jörg Jenatsch, Assassinated 1639 in Chur, Switzerland

**DOI:** 10.1371/journal.pone.0168014

**Published:** 2016-12-28

**Authors:** Martin Haeusler, Cordula Haas, Sandra Lösch, Negahnaz Moghaddam, Igor M. Villa, Susan Walsh, Manfred Kayser, Roger Seiler, Frank Ruehli, Manuel Janosa, Christina Papageorgopoulou

**Affiliations:** 1 Institute of Evolutionary Medicine, University of Zürich, Zürich, Switzerland; 2 Institute of Anatomy, University of Zürich, Zürich, Switzerland; 3 Zurich Institute of Forensic Medicine, University of Zürich, Zürich, Switzerland; 4 Department of Physical Anthropology, Institute of Forensic Medicine, University of Bern, Bern, Switzerland; 5 Institute of Geological Sciences, University of Bern, Bern, Switzerland; 6 Centro Universitario Datazioni e Archeometria, Università di Milano Bicocca, Milano, Italy; 7 Department of Biology, Indiana University Purdue University Indianapolis (IUPUI), Indianapolis, Indiana, United States of America; 8 Department of Genetic Identification, Erasmus MC University Medical Centre Rotterdam, Rotterdam, The Netherlands; 9 Archäologischer Dienst Graubünden, Chur, Switzerland; 10 Laboratory of Anthropology, Department of History and Ethnology, Demokritus University of Thrace, Komotini, Greece; University of Florence, ITALY

## Abstract

Jörg Jenatsch, a leading freedom fighter during the Thirty Year’s War in Graubünden, Switzerland, was assassinated on carnival 1639. Jenatsch’s controversial biography and the unclear circumstances of his death inspired the formation of various legends, novels and films. In 1959, a skeleton discovered in the cathedral of Chur with remains of wealthy baroque clothing was tentatively attributed to Jenatsch. Here, we reassess the skeleton based on a new exhumation. Our multidisciplinary analysis and the head injuries are consistent with reports of the eyewitnesses of the crime, demonstrating that Jenatsch was killed from behind with a semi-sharp implement, supposedly an axe, as well as by a blow with a broad-surfaced object. Moreover, our facial reconstruction closely matches an oil portrait of Jenatsch, and the HIrisPlex system applied to DNA-extracts from the femoral bone reveals brown eye and dark brown hair colour, which coincides well with the portrait, too. Finally, isotope analysis of the femoral bone and a molar support Jenatsch’s high social status, luxury diet and a high mobility in the last decade of his life. This multidisciplinary approach thus reinforces personal identification and provides additional insight into the life of this important historic person beyond written resources.

## Introduction

Jörg (Georg) Jenatsch (1596–1639) was a Protestant pastor and a famous political and military leader in the Bündner Wirren (Confusion of the Leagues, 1618–1639) during the Thirty Years’ War in what is now the Swiss canton of Graubünden [1–4]. To preserve the independence of his home country, the Three Leagues of Graubünden, and its control over the strategically important Valtellina and Chiavenna, Jenatsch entered into changing alliances with France-Venice and the Spanish-Austrian Habsburg Monarchy and even converted from Protestantism to Catholicism. His divisive life and violent death were immortalized by Conrad Ferdinand Meyer in the popular novel “Jürg Jenatsch” [5] and by Daniel Schmid in the movie “Jenatsch)” [6] as well as in many biographies [1–3].

**Fig 1:**
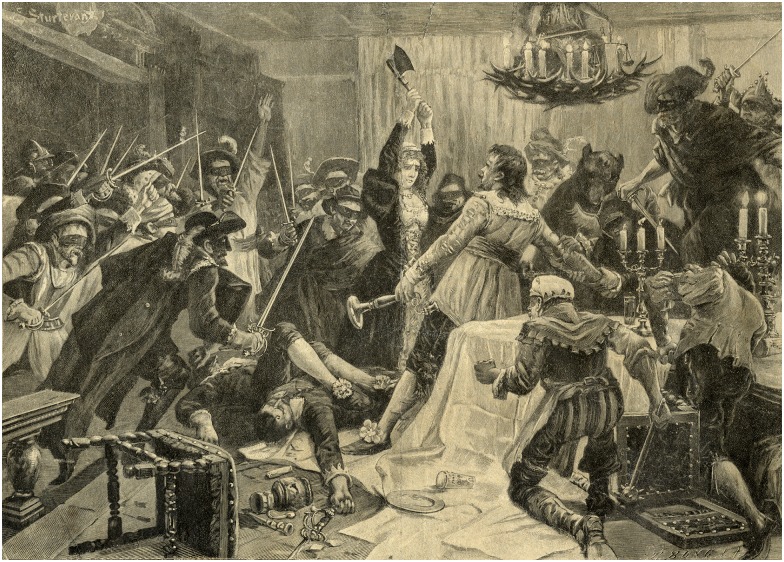
The assassination of Jörg Jenatsch during carnival, 24^th^ of January 1639, in the tavern “Zum staubigen Hüetli” in Chur. Wood engraving by R. Bong after a painting by E. Sturtevant, probably late 19^th^ century, Rätisches Museum Inv. No. H1972, 1748. The painting is based on the erroneous account in the novel of C.F. Meyer that the imaginary Lucrezia von Planta revenged her father’s death using the same axe with which Jenatsch and his fellows killed Pompeius von Planta in 1621 (ref. [7]).

**Fig 2:**
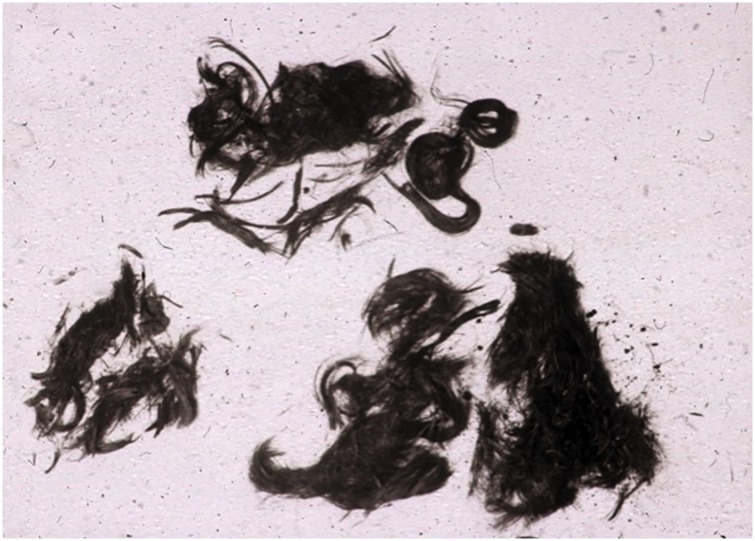
Tufts of blood-soaked brownish-black shoulder-long hair from the back of the head and both temples, isolated during the first exhumation in 1959. The hair colour was determined by Hug [11] as No. W/X according to the colour scheme in ref. [12], the hair shape as narrow-waved (kymatotrich) according to ref. [13].

On the 24^th^ of January 1639, Jenatsch was assassinated with an axe while celebrating carnival in a tavern in Chur (Fig 1). It was never completely resolved who of Jenatsch’s many political and personal enemies were the masked murderers, though blood vengeance might have been a leading motive [7–9]. Twelve hours later, Jenatsch was buried in the Chur cathedral below the former organ. The exact location of his grave, however, fell into oblivion when the gravestones of the cathedral floor were rearranged during a renovation in the mid-19^th^ century [10]. A first quest for Jenatsch in 1959 by Erik Hug (1911–1991) resulted in the recovery of two skeletons in the vicinity of the presumed original location of the gravestone, one of which was initially assigned to Jenatsch [11]. The lack of evidence for perimortal trauma in that skeleton, however, gave rise to doubt. Further excavations disclosed a third skeleton that better fitted the expectations. Although the rear part of its skull was badly weathered, both temples showed evidence for extensive blunt and semi-sharp head trauma, respectively. Moreover, the remains of wealthy clothing in baroque style and medium-length dark hair (Fig 2) were obviously soaked with blood, which was supported by positive benzidine and leucomalachit reactions [11].

While the archaeological remains of the 1959 excavation were turned over to the treasure vault of the Chur cathedral, the bishop wanted the skeleton to be reburied two years later. Unfortunately, the extensive documentation of the excavation and the following analyses was lost upon the death of Erik Hug in 1991. After a long adventurous quest led by one of us (MJ), the documentation was recently rediscovered [10], and a new exhumation was arranged in 2012 in order to address unsolved questions and to reanalyse the skeleton with modern technologies [4]. For two days it was possible to examine the skeleton macroscopically, by conventional radiography and computed tomography as well as to take a bone sample of the femoral shaft and to extract two teeth for further analyses, before the skeleton had to be reburied again.

In a previous genetic analysis we studied the Y-STRs and Y-SNPs from the bone sample [14]. Unexpectedly, we found that the skeleton and three living family members 14 generations apart were discordant at three of 23 Y-STR loci (DYS456, DYS458 and DYS635), though they carried the same Y-SNP-haplogroup. Nevertheless, biostatistical evaluation of the data suggested a high likelihood that the skeleton belonged to the Jenatsch family. In this study we present the multidisciplinary evidence to whether the skeleton indeed belongs to the controversial freedom fighter Jörg Jenatsch. We retrieve information on the externally visible characteristics (EVCs), namely eye and hair colour by using the HIrisPlex system [15]. We compare the results to the hair tufts recovered during the 1959 excavation and to an oil painting of Jörg Jenatsch [4] made three years before his death. This portrait is also used for comparison with a facial reconstruction based on Jörg Jenatsch’s presumed skull. In addition, stable isotope analysis provides more information about Jenatsch’s dietary habits, mobility and social status.

## Materials and Methods

### Morphological analysis

Sex, age and stature determination followed standard methods [16,17]. Conventional radiographs and computed tomography of the skeleton have been made at the Radiology Department of the Cantonal Hospital Chur. The CT scans were done with a low dose protocol and 1 mm slice thickness on a Toshiba Aquilion 5634. In addition, the skull was scanned using the pyramid/petrous bone protocol (tube voltage 120 kV, tube current 50 mA, slice thickness 0.5 mm, pixel spacing 0.431 mm /0.431 mm, pixel depth 16 bits). This high-resolution scan was used to calculate a 3D surface model of the skull. The mandible was reconstructed by mirror imaging in Rhinoceros 5.0. The individual fragments of the right-sided impression fracture were reoriented in virtual space to restore their original position and the plastically deformed posterior portion of the right parietal was realigned using bilateral symmetry.

### Facial reconstruction

The skull was then printed on a 3D printer using the Color Jet printing technology, which deposits layer by layer a liquid binder onto a plastic powder at a layer thickness of 0.1 mm. The missing parts were modelled in wax onto this copy of the skull. Fifty-two markers for soft tissue thickness were placed according to Helmer [18,19]. Soft tissue thickness was based on the mean values of 40 to 49-year-old white European males. Facial reconstruction followed Richard Neave’s Manchester method [18,20]. Only after finishing the facial reconstruction, it was compared to the portrait of Jörg Jenatsch.

Several historic copies of an oil portrait of Jörg Jenatsch are known. Most differ slightly in facial proportions, the inscription and details of the clothes, but all show the year 1636, i.e. three years before Jenatsch’s death. The original painting was thought to have been lost after it was returned by the Rätisches Museum, Chur, to its owners in 1935. However, a recent comparative-radiological examination demonstrated that the Jenatsch portrait displayed in the Swiss Embassy in Paris must be the original [4]. We compared the facial proportions of this portrait with those of the finished facial reconstruction. We also used it as reference for the hairstyle of our reconstruction and and for the result of the HIrisPlex genotyping of the skeleton.

### Genetic analysis

DNA extraction was performed on a bone sample from the left femoral diaphysis (sample A) as described in ref. [14]. The Y-chromosome analyses of that study showed that the DNA was degraded, but sequences of up to 400 bp could successfully be analysed. We adhered to the accepted guidelines for aDNA work, including extraction in a dedicated aDNA laboratory, isolation of pre- and post-PCR areas, appropriate negative controls and the analysis of multiple PCRs, though from a single extract [14,21,22]. HIrisPlex genotyping followed the protocol of ref. [15]. The maximum possible DNA input amount of 4 μl was tested in replicate PCR and SBE reactions in the laboratories of the Department of Forensic Molecular Biology at Erasmus MC University Medical Centre Rotterdam and the Zurich Institute of Forensic Medicine. Thermocycling was performed on GenAmp PCR System 9700 thermocyclers (life technologies). Using two 3130xl Genetic Analyzers, 1 μl of the cleaned product was run with POP-7/POP-4 and with the following parameters: 2.5/2 kV, 10/11s injection voltage, 500/1000s run time at 60°C. Raw data were analysed with Genemapper Software (v3.7/ID-X v4.1, Life technologies). A threshold of 50 RFU (relative fluorescence units) was used for peak detection. An additional piece from the left femoral diaphysis (sample B) leftover from isotopic analysis was available for DNA analysis. Although it was not treated DNA safe and minor contamination from a staff member (person C) was detected by STR profiling it was subjected to HIrisPlex genotyping together with person C.

The recommendations for reliable SNP genotyping from historical and low-quality samples [23] were applied to bone sample A. Following Taberlet et al. [24], the experimentally derived dropout rate was used to calculate the amount of repetition necessary for correct results at the 99% certainty level. Genotypes were accepted as ‘true’ if both alleles of heterozygous genotypes were seen at least twice, and if homozygous genotypes were replicated at least three times.

### Isotope analysis

Part of the bone sample B from the femoral diaphysis was used for stable isotope analysis followed modified extraction protocols of Ambrose [25] and Longin [26]. 500 mg bone powder was treated with 1 mol/l HCl to dissolve the bone mineral. The sample was treated with 0.125 mol/l NaOH to remove humic acids and gelatinized in 0.001 mol/l HCl at 90C for 17 h. After filtration the sample was freeze-dried. The lyophilized collagen was weighed in tin capsules (3x). Stable isotope ratios of carbon (^13^C/^12^C), nitrogen (^15^N/^14^N) and sulphur (^34^S/^32^S) were analysed by isotope ratio mass spectrometry (IRMS) at Isolab GmbH, Schweitenkirchen, Germany. The mean of three measurements was used. The data are reported in δ-notation in per mil (‰) and measured relative to international standards, Vienna Pee Dee Belemnite (VPDB) for carbon, Ambient Inhalable Reservoir (AIR) for nitrogen and Canyon Diablo Troilite (CDT) for sulphur [27]. The analytical error was internally determined to 0.1‰ for δ^13^C, 0.2‰ for δ^15^N and 0.3‰ for δ^34^S.

Bone constantly remodels during life. The turnover rate of collagen at the femoral midshaft decreases from, on average, 10–30% per year during the adolescent growth spurt to 3% per year at age 20 and 1.5% per year at age 80 in adult males [28]. The relatively slow turnover rate of collagen from the femoral cortex in adults is therefore thought to isotopically reflect an individual’s diet over at least 10 years prior to death, but it may include a substantial portion of collagen produced during adolescence [28].

Carbon stable isotope ratios (δ^13^C) in bone collagen show dietary enrichment of C_3_-plants, such as wheat and barley, or C_4_-plants, e.g. millet [29]. The δ^15^N value provides information about the trophic level and thus reflects the intake of animal protein [30–33]. The δ^34^S ratio in bone collagen is passed along the food chain with a small fractionation of approximately -1‰. As δ^34^S values vary in different geographical regions and geological conditions, stable sulphur isotope data also provide information about migration of individuals [34,35]. The ^87^Sr/^86^Sr and ^207^Pb/^206^Pb isotope ratios are used to obtain information about mobility [36–39].

The analyses of the strontium and lead isotope ratios were done at the Institute of Geological Sciences, University of Bern. Samples of the tooth enamel from the crown and of the dentin from the root of a second right mandibular molar (tooth 37) were separated. A total of 43 mg tooth enamel and 72 mg dentin was obtained by a miniature rotating diamond-blade saw. The samples were dissolved in 14.4 mol/l HNO_3_ and 30% H_2_O_2_. The oxidizing mixture removed all remaining organic matter from the solution. The separation of Sr and Pb from the Ca matrix on Sr-Spec^™^ resin followed the protocol adapted from ref. [40]. The sample was loaded as nitrate onto the miniaturized columns containing Sr-Spec^™^ resin. Ca and other matrix elements were flushed by 1 mol/l nitric acid while both Sr and Pb were retained on the resin. Sr was then eluted with 0.01 mol/l nitric acid while Pb was still retained; finally, Pb was eluted with 6 mol/l HCl. Isotopic compositions were measured on a Nu Instruments^™^ plasma-source multicollector mass spectrometer using established protocols for Sr (ref. [41]) and Pb (ref. [40]).

## Results

### Morphological analysis

**Fig 3:**
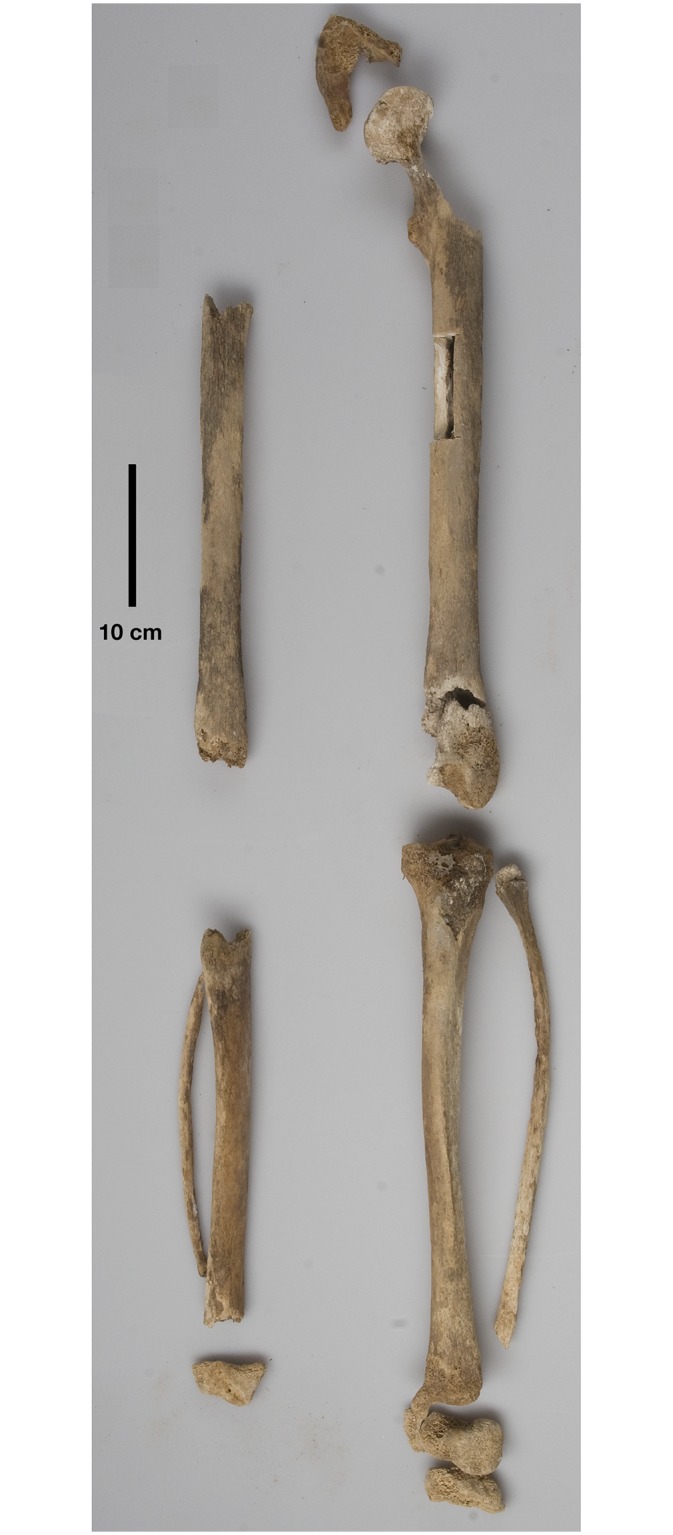
The preserved bones of the lower extremities. The 7 x 2 cm large defect in the left femoral shaft resulted from DNA sampling. Scale bar 10 cm.

**Fig 4:**
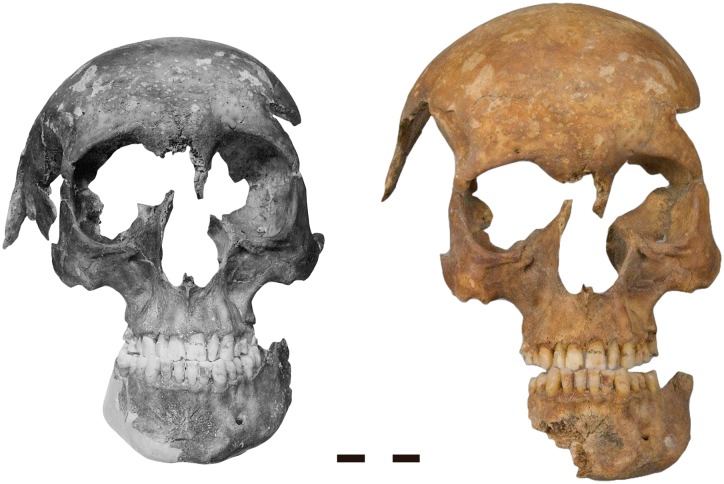
Preservation of the skull during the first exhumation compared with that of the second exhumation. Left: condition in 1959, Right: condition in 2012. Note the progressive weathering of all edges. On the right, the mandible is not in occlusion, and the neurocranium is reconstructed in a different orientation to the viscerocranium than in 1959. Moreover, the fragments of the spider web fracture of the right temple have not been realigned. Scale bar 3 cm.

The skeleton was in a severely weathered condition during the 2012 excavation. It preserved the anterior part of the skull and a few postcranial bones, including parts of the right ulna shaft, the lunate and capitate bones of the right wrist, the left pubic symphysis, and fragments of both femora, tibiae, fibulae and calcanei, and of the left talus (Fig 3). Some bones, including a trapezoid, seem to have disappeared in 2012 compared to Erik Hug’s 1959 notes, and a comparison of the bones with their 1959 photographs suggested that chemical weathering rapidly progressed in the 53 years between the two exhumations (Fig 4). This might be attributable to the unfortunate choice of sand for reburial, which favoured washing-off of brushite and other soluble bone decomposition products [42]. It was therefore no longer possible to check Hug’s measurements of femur length. The best preserved bone was the left tibia, which had a greatest length of 390 mm (Martin [13] measurement 1a) and a lateral length of 380 mm, respectively (Martin [13] measurement 1). This implied a stature of between 169 cm (regression formula for southern Europeans [43]) and 173 cm (with body proportions of middle and northern Europeans [43]).

The skull preserved mainly the facial bones together with the frontal bone, the anterior regions of the parietals, the zygomatic bones and the adjacent parts of the temporal bones. The sexual characteristics clearly indicated a male individual, which was supported by a very robust mandible with a square chin, a narrow subpubic angle in the hipbone fragment and an absent ventral arc.

**Fig 5:**
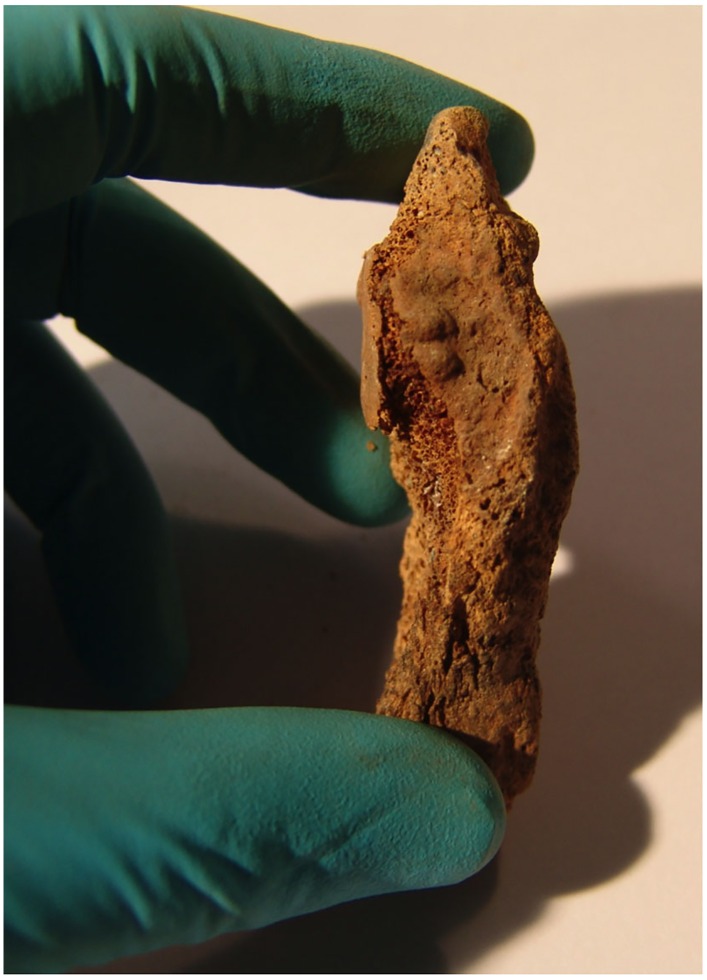
Symphyseal face of the left pubic bone fragment. Note the well-marked ridges and the ventral and dorsal margins showing rim formation, consistent with stage II according to Acsádi and Nemeskéry [16].

The symphyseal face of the pubic bone was crucial for age determination. Ridges were still well marked while the ventral and dorsal margins showed beginning rim formation (Fig 5). This would classify as stage II according to Acsádi and Nemeskéry [16]. Computed tomography of the proximal femur suggested stage II for the rarefication of the trabecular structure, while assessment of the endocranial sutures indicated complete closure (stage V). In combination [16], these three features would suggest a mean age of 43.3 years ± 3.3 years, which exactly corresponds to the 43 years of Jörg Jenatsch’s age at death. Due to weathering, however, attribution of the skeleton to the 40–50 years age group would probably be more conservative.

**Fig 6:**
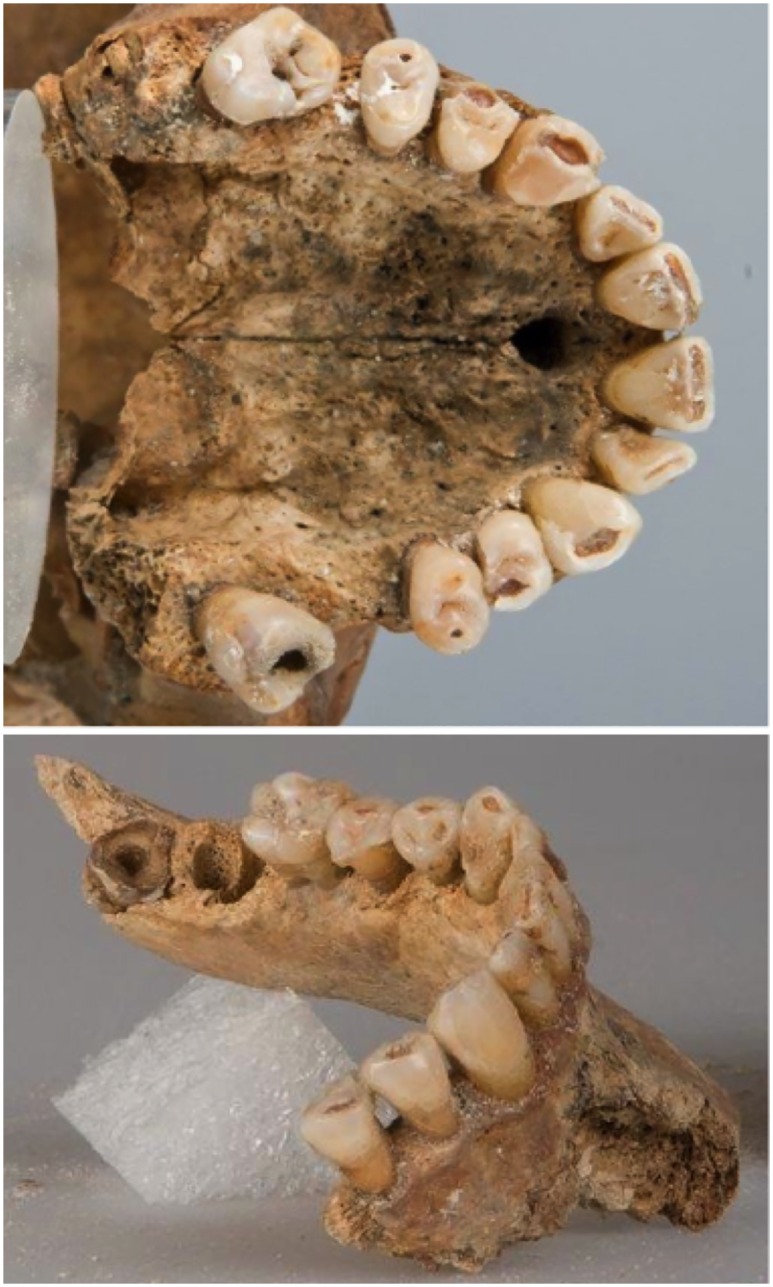
Occlusal view of the maxilla (top) and right oblique view of the mandible (bottom).

Unfortunately, tooth cementum annulation could not be assessed. Tooth abrasion, as another possible age indicator appeared unusually high for an age of 43 years compared to modern human standards, although not as high as in some prehistoric populations [44] (Fig 6).

**Fig 7:**
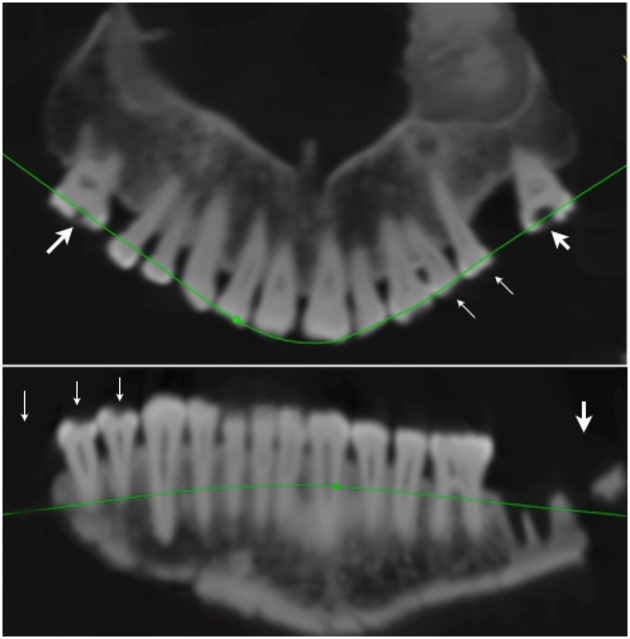
CT based panoramic radiograph of the maxillary (top) and mandibular dentition (bottom). Two teeth have been extracted before radiographic examination (right first and left second mandibular molar), while the left mandibular wisdom tooth, whose clinical crown was completely destroyed, was not in its alveolus, and both maxillary first molars have been lost intra vitam. Radiographically visible deep carious lesions are shown by large arrows; small carious lesions that are only visible macroscopically are shown by small arrows.

Teeth can, however, also hint at a person’s living conditions. Thus, both maxillary first molars (ISO notation: teeth 16 and 26) have been lost intra vitam (Fig 6). Their loss is probably due to caries as implied by marked caries of the neighbouring teeth. In the mandible, the right wisdom tooth (tooth 38) was lacking, perhaps due to postmortem tooth loss, while the left second and the right first mandibular molars (teeth 37 and 46) have been extracted for DNA and isotope analysis, respectively, before radiological examination. The CT based panoramic radiograph (Fig 7) and macroscopic examination confirmed that eight teeth (or ten if the two intravitally lost upper molars are included) were affected by caries. Nevertheless, the dentition seems to have been fully functional with a robust periodontium in the anterior region.

### Skull trauma

**Fig 8:**
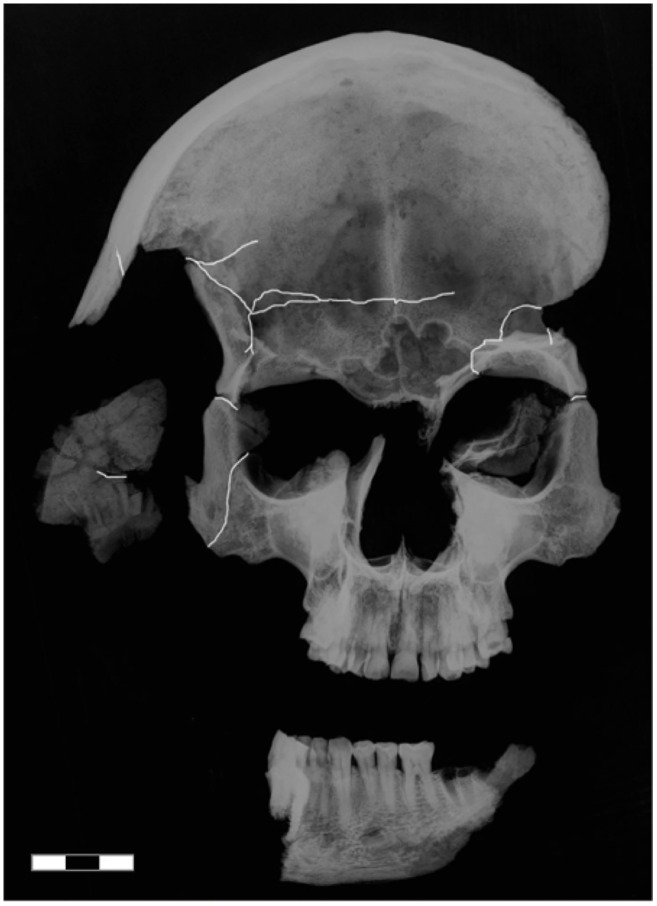
Anteroposterior radiograph of the skull. Fracture lines are emphasized with white lines. Scale bar 3 cm.

**Fig 9:**
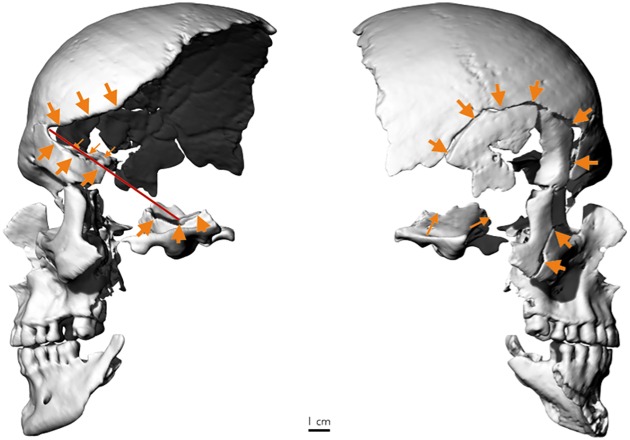
CT based three-dimensional reconstruction of the skull. (a) Left lateral view, showing a 7 cm long wedge-shaped notch that runs from the region above the left eye to the left ear. Small arrows indicate the fracture of the inner side of anterior cranial fossa. (b) Right lateral view. The extensive impression fracture of the right temple is well visible (arrows).

Both sides of the skull show evidence for extensive trauma. The anteroposterior radiograph of the skull reveals a fracture pattern typical for fresh bone that cannot be explained by taphonomic processes (Fig 8). The right temple shows a large, almost circular impression fracture with a diameter of over 7 cm and multiple bone fragments. Comparable to a spider web fracture, the longest fracture line radiates from the right supraorbital region, crosses the frontal bone and ends in the left supraorbital region. This is characteristic of blunt force trauma with a broad surfaced object [45]. While the location of the fracture is not typical for a fall [45], the preserved fragments do not allow inferring the direction of the blow nor the type of the object involved. On the left temple, an approximately 4 cm long wedge-shaped notch is visible, from where fracture lines run into the superior margin of the left orbita. A three-dimensional reconstruction of the skull suggests that the notch continued for about 7 cm to the left ear (Fig 9). In the same plane with this line lies also the fracture of the inner side of the anterior cranial fossa. Although weathering has smoothed the edges, this pattern is compatible with a deep cut of a 7 cm broad, semi-sharp chopping implement. Unfortunately, no fragments of the fracture system of the left temple are preserved. Moreover, because the fracture systems of both temples do not cross each other, the temporal sequence of the injuries cannot be inferred.

### Facial reconstruction

**Fig 10:**
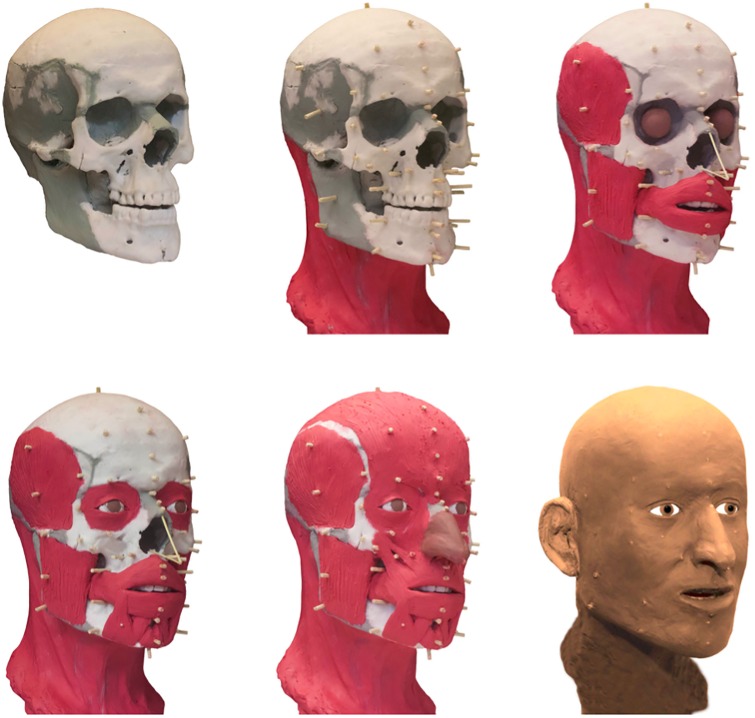
Different stages of the facial reconstruction.

**Fig 11:**
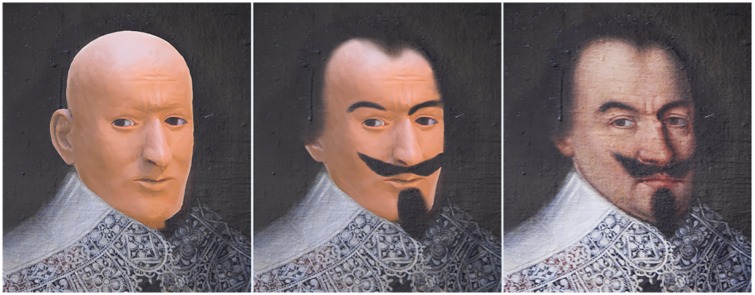
Comparison of the facial reconstruction with the portrait of Jörg Jenatsch. A) Raw facial reconstruction, performed without influence of the portrait. B) The same hairstyle superimposed on the facial reconstruction as in the portrait. C) Original portrait of Jörg Jenatsch (Courtesy of Swiss Embassy in Paris, France).

Fig 10 shows the different stages of the facial reconstruction. A superposition of the reconstruction onto the portrait of Jörg Jenatsch demonstrates a surprisingly good match of the facial proportions (Fig 11). Frontal breadth and height, distance and position of the eyes, ears, length and position of the nose, mouth and chin correspond perfectly. Only the bump at the root of the nose could not be inferred from the preserved facial skeleton and the cheeks are less bulging than on the portrait.

### Genetic analysis of eye and hair colour

To predict eye and hair colour from bone DNA, six replicate HIrisPlex analyses (covering all 24 SNPs) and one singleplex analysis (SNP20, rs12913832, HERC2) were performed with the extract from bone sample A. SNP 20 is the most important SNP to predict brown versus non-brown eye colour and also important to differentiate blond/brown/black hair colour [15]. Six partial profiles were obtained from the replicate analyses and a clear result from the singleplex (Table 1). The replicate analyses for the 24 SNPs generally yielded coinciding results, but as expected with ancient DNA samples some drop ins and drop outs occurred. Particularly, five SNPs of bone sample A (i.e., SNPs 15 (rs4959270, EXOC2), 17 (rs1042602, TYR), 19 (rs2402130, SLC24A4), 22 (rs12896399, SLC24A4) and 23 (rs1393350, TYR)) showed ambiguous results. Possible options for the handling of these five SNPs included (Table 2): (1+2) omit problematic genotypes (including or excluding SNP23), (3) follow replicate rule, (4) replicate rule and ‘additional information for SNP23’. The results for SNP 23 provided by bone sample B (CT) and person C (CC) argued for a heterozygous genotype in bone sample A. The eye and hair colour prediction probabilities were calculated for all four options, and resulted in almost identical probability values. We used the enhanced Irisplex eye colour prediction model based on a model-underlying reference database of genotypes and phenotypes from >9000 individuals and the enhanced HIrisPlex hair colour prediction model based on >1600 individuals as available under http://hirisplex.erasmusmc.nl. The most likely eye colour was concluded using the category with highest probability [46] and the most likely hair colour using the hair colour prediction guide [15]. Based on the results from bone sample A, the most probable eye colour of the analysed individual was brown, with a probability of 97–98% while the probabilities for blue and intermediate colours where with 0.03–0.1% and 1.4–2.3% considerably lower. The most probable hair colour was dark brown with a probability of 62% for brown, while the probabilities for blond, black and red were with 20%, 17% and 0.05–0.08% much lower. The probabilities for dark hair shade were 53–56% compared to 43–46% for light hair shade.

**Table 1:** Hair and eye colour analysis based on the HIrisPlex system, results from bone samples A and B, and person C.

Pos.	SNP	ma/mi	Person C			Bone B*						Bone A**							eye+hair colour predicition tool					
																			options	best	1	2	3	4
1	N29insA_(DU)snF	C/A	C	C	C	-	-	-	C	-	C	(C)	(C)	C	-	C	C				0	0	0	0
2	rs11547464_(H)snF	G/A	G	G	G	-	G	G	G	-	-	G	G	G	G	G	G				0	0	0	0
3	rs885479_(BD)snR	C/T	C	C	C	-	C	C	C	-	-	C	(C)	C	(C)	C	C				0	0	0	0
4	rs1805008_(FU)snF	C/T	C	C	C	-	C	C	C	-	-	(C)	(C)	C	-	C	C				0	0	0	0
5	rs1805005_(FU)snF	G/T	GT	GT	GT	-	-	-	G	G	G	-	G	G	-	G	G				0	0	0	0
6	rs1805006_(FU)snF	C/A	C	C	C	-	-	-	C	C	C	-	C	C	-	C	C				0	0	0	0
7	rs1805007_(T)snF	C/T	C	C	C	-	C	C	C	-	-	C	-	C	-	C	C				0	0	0	0
8	rs1805009_(T)snF	G/C	G	G	G	-	G	G	G	G	G	G	G	G	G	G	G				0	0	0	0
9	Y152OCH_(FU)snF	C/A	C	C	C	-	C	C	C	-	-	C	-	C	-	C	C				0	0	0	0
10	rs2228479_(T)snF	G/A	G	G	G	-	-	-	G	G	G	-	G	G	-	G	G				0	0	0	0
11	rs1110400_(T)snF	T/C	T	T	T	-	-	T	(T)	-	-	(T)	-	T	-	T	-				0	0	0	0
12	rs28777_(july)snF	A/C	A	A	A	-	A	-	-	A	A	A	A	A	-	A	A				0	0	0	0
13	Rs16891982_(T)snF	G/C	G	G	G	G	-	G	G	-	G	-	G	G	G	G	-				0	0	0	0
14	rs12821256_(T)snR	A/G	A	A	A	A	AG	-	G	A	G	AG	AG	AG	(A)G	AG	AG				1	1	1	1
15	rs4959270_(T)snF	C/A	CA	CA	CA	C	A	A	C	C	-	A	C	C	-	C	-		0 or 2	0	-	-	0	0
16	rs12203592_(T)snF	C/T	C	C	C	-	-	C	C	C	C	C	C	C	-	C	-				0	0	0	0
17	rs1042602_(T)snR	G/T	T	T	T	G	GT	G	GT	G	GT	G(T)	GT	GT	G	G	T		0, 1 or 2	1	-	-	1	1
18	rs1800407_(T)snF	G/A	G	G	G	G	G	G	G	G	G	-	G	G	-	G	G				0	0	0	0
19	rs2402130_(T)snF	A/G	GA	GA	GA	A	A	A	-	AG	-	A	GA	A	-	A	A		0 or 1	0	-	-	0	0
20	rs12913832_(T)snR	C/T	C	C	C	T	-	-	CT	T	-	T	T	CT	T	T	T	T***			2	2	2	2
21	rs2378249_(T)snR	T/C	T	T	T	T	T	-	T	T	T	T	T	-	-	T	T				0	0	0	0
22	Rs12896399_(T)snF	T/G	TG	TG	TG	T	T	T	T	TG	G	TG	T	T	T	T	TG		0 or 1	0	-	-	0	0
23	Rs1393350_(DU)snR	C/T	C	C	C	CT	-	-	CT	CT	CT	T	C	C	(C)	T	CT		0 or 1	1	-	1	0	1
24	rs683_(H)snR	T/G	TG	TG	TG	T	-	TG	TG	T	T	TG	G	(T)G	-	TG	TG				1	1	1	1

- = no result, () = peak height below 50 RFU (relative fluorescence unit).

* Bone B is contaminated with Person C. These results were only used for the interpretation of the SNP 23 data.

** For the interpretation of results for bone sample A, four possible options for five problematic SNPs were considered and used for probability prediction calculations.

*** Singleplex

**Table 2:** Prediction probabilities for hair colour categories and shade, as well as eye colour, for the four options of bone sample A.

	eye colour						hair colour								hair shade		
Option	PBlue	Pintermediate	PBrown	AUC_ Loss_Blue	AUC_Loss_ Intermediate	AUC_ Loss_Brown	PBlond	PBrown.1	PRed	PBlack	AUC_ Loss_Blond	AUC_ Loss_Brown.1	AUC_ Loss_Red	AUC_ Loss_Black	PLight	PDark	AUC_ Loss_Shade
1	0.00089	0.02070	**0.97842**	0.01556	0.04023	0.00858	0.20087	**0.62327**	0.00084	0.17501	0.00452	0.00475	0.00413	0.00268	0.46378	**0.53622**	0.00233
2	0.00104	0.02271	**0.97626**	0.01025	0.02867	0.00527	0.20087	**0.62327**	0.00084	0.17501	0.00452	0.00475	0.00413	0.00268	0.46378	**0.53622**	0.00233
3	0.00028	0.01445	**0.98528**	0	0	0	0.19448	**0.62655**	0.00052	0.17845	0	0	0	0	0.43889	**0.56111**	0
4	0.00042	0.01787	**0.98171**	0	0	0	0.19448	**0.62655**	0.00052	0.17845	0	0	0	0	0.43889	**0.56111**	0

The loss to AUC (area under the curve) is given for the options where SNPs are missing.

### Isotope analysis

The results of the stable isotopes analysis are presented in Table 3. The data follow the collagen quality criteria [25,47] where collagen should have more than 1% in proportion to the dry weight, the molar C/N relation should range between 2.9 to 3.6 and the %C and %N should not strongly diverge from recent collagen values. The concentration of strontium was measured to 205 ppm for the enamel and 83 ppm for the dentin sample.

**Table 3:** Data of the isotope analysis from femur bone collagen (C, N, S), tooth enamel and dentin samples (Sr, Pb), mean and standard deviations.

Analysis	Bone	Enamel	Dentin
%coll	10.3		
C (%)	47.8		
N (%)	17.6		
S (%)	0.1		
C/N mol	3.1		
δ^13^C	19.6 (±0.1)		
δ^15^N	10.3 (±0.2)		
δ^34^S	6.1 (±0.3)		
^87^Sr/^86^Sr		0.711034 (±0.000036)	0.709104 (±0.000031)
^207^Pb/^206^Pb		0.84412 (±0.00005)	0.84672 (±0.00005)

## Discussion

The combined anthropological, traumatological, genetic and archaeometric evidence suggests a high probability that the skeleton from the Chur cathedral is in fact that of Jörg Jenatsch.

First, the graves’ position below the organ of the cathedral, the wealthy non-clerical baroque clothing from the first half of the 17^th^ century, as well as age and sex determination of the skeleton (suggesting a ca. 43-year-old male according to the combined method of Acsádi and Nemeskéry [16], or more conservatively a male between 40 and 50 years) perfectly concur with the individual data of Jörg Jenatsch.

Second, the evidence of the skull trauma is consistent with the records of the night of the murder. The eyewitnesses of the assassination testified that Jenatsch was celebrating carnival when five masked men entered the tavern [7,8,48]. Their leader was said to have saluted Jenatsch by taking his right hand while pulling a pistol out of his costume. The bullet missed though or wounded him only superficially, whereupon a second man felled Jenatsch with an axe and, while already lying on the floor, other men smashed his head and body with a riding hammer. Unfortunately, traces of spent gunpowder could not be recovered from the exhumed clothes during the 1959 analysis [11]. Moreover, accelerated weathering after the first reburial has smoothed the sharp edges of the skull fractures that were observed at that time [11]. Nevertheless, the pattern on the left temple indicates semi-sharp force trauma [45] that is consistent with an approximately 7 cm wide blade of a chopping implement that could have been the axe reported by the eyewitnesses. The position and orientation of the injury suggests that the blow occurred from behind while Jenatsch was either standing or lying, probably by a left-hander [49]. On the other hand, the spider web fracture of the right temple is characteristic for blunt force by a broad-surfaced object [45]. Unfortunately, the direction of the impact cannot be inferred due to weathering. Because the fracture systems of both temples do not cross each other, the temporal sequence of the injuries cannot be determined, but both injuries must have led to immediate death.

Third, the soft-tissue reconstruction of the skull shows accordance with the oil portrait of Jörg Jenatsch. Only the cheeks are less puffy in the reconstruction. Although baroque paintings are not necessarily photo-realistic, as the patrons often asked for idealised portraits [4], this might reflect a stouter body shape of Jenatsch than the average of the individuals whose soft tissue thickness was used as reference for facial reconstruction. In fact, the analysis of the jacket found in 1959 suggested a waist circumference of 122 cm [11]. In a modern Scottish population, a waist circumference of greater than 102 cm correlated with a BMI of over 30 kg/m^2^ corresponding to obesity [50]. This would imply that Jenatsch was very well nourished also if the maximum stature of 175 cm estimated from tibia length is taken into account. Nevertheless, it is difficult to determine how high the sensitivity and specificity of a facial reconstruction actually is. A study that compared the superimposition of three skulls with 100 photographs of different faces in television quality found 8.5% incorrect pairings for the frontal view [51]. If in addition also a lateral photograph was compared to the skull, the rate of false matches dropped to 0.6%. Modern computer based digital techniques might perhaps further increase the accuracy of the superimposition [52], but also if morphological details, e.g., the shape of the nose, are taken into account [53].

Forth, the HIrisPlex system [15] predicts brown eyes and dark brown hair for Jörg Jenatsch, which perfectly matches the 1636 portrait. From replicate analyses an almost complete HIrisPlex profile could be generated. Several options were considered for the five problematic SNPs 15, 17, 19, 22 and 23. The prediction probabilities were very similar for all options, meaning that genotypes in question at these five problematic SNPs together with the genotypes obtained at the remaining 19non-problematic SNPs did not contribute substantially to eye and hair colour prediction. The major impact in determining whether the eye colour will be brown versus non-brown comes from SNP20 (rs12913832, HERC2) [54], while SNPs 16 (rs12203592, IRF4) and 20 (rs12913832, HERC2) contribute most to blond/brown/black hair discrimination [15]. Our results from the bone sample for these two SNPs were concordant and reproducible, suggesting that Jenatsch had indeed brown eyes and dark brown hair. Hair tufts recovered at the first exhumation in 1959 in the skull region have also been described as brownish black [11]. Unfortunately, this hair sample is lost today. Inferring intra vitam colour from ancient hair samples is difficult as taphonomic processes including deposits of (soil) particles and oxidation of keratin and melanin pigments can change hair colour and structure [55]. Jenatsch’s hair was apparently soaked with blood [11], and oxidized haemoglobin might therefore have blackened the hair.

A similar approach has recently been successful in the identification of King Richard III of England (1462 to 1485) [56–59]. Eye and hair colour of Richard III were established by applying the HIrisPlex system as well, which revealed blue eyes and blond hair with probabilities of 96% and 77%, respectively. This allowed the conclusion that King Richard III most likely had blue eyes and blond to dark blond or light brown hair [59] (depending on whether or not his blond hair colour as child had darkened during adolescence, which cannot be detected with the HIrisPlex system [15]). In fact, a portrait painting made about 25 years after his death (no painting is available from his lifetime) shows him with light eyes and light brown hair [59].

Fifth, the identification of the skeleton as that of Jörg Jenatsch is also supported by the analysis of Y-STR and Y-SNP profiles [14]. The skeleton and three living family members carried the same Y-SNP haplogroup, but were discordant at three of 23 Y-STR loci (Y-STR loci DYS456, DYS458 and DYS635). Conservative biostatistical evaluation of the Y-STR data showed that the probability of a one-repeat mismatch at exactly three of 17 loci in one of 12 meioses was 20 times more likely than the probability that two Yfiler haplotypes randomly drawn from a general European population exhibit exactly three mismatches [14]. Our Y-chromosomal analysis therefore strongly suggested that the skeleton is that of Jörg Jenatsch.

Sixth, reconstructions of life history parameters demonstrate that the skeleton must have belonged to an individual of high social status. Although Jenatsch was the son of an ordinary pastor, his stature of 169 to 173 cm is above the estimated mean stature of the slightly earlier, but geographically close population from Tomils (11^th^ to 15^th^ century AD, 164.1 cm ± 5.2 cm) [60]. This indicates a comparatively good nutrition and sanitation during his growth and an above-average socioeconomic status [61]. This is supported by the absence of Harris lines in the radiographs of the tibiae and the absence of linear enamel hypoplasias of the teeth [62].

The high percentage of teeth affected by caries provides a less evident indication for Jörg Jenatsch’s socioeconomic status. Although caries prevalence is often considered to be increased in low social classes [63], it might also reflect a relatively high proportion of carbohydrates in the diet and perhaps even consumption of sugar canes and other cariogenic food that was less accessible to the average contemporary at the beginning of the 17^th^ century. This is in agreement with the observed relatively high tooth abrasion that suggests a diet rich in flour ground with millstones. Although he exhibits a high frequency of caries and ante-mortem tooth loss he did not suffer from abscesses that are very frequent in earlier populations from the same geographical region such as the medieval Tomils (11^th^-15^th^ century AD) [60] This might indicate a relatively costly treatment by a barber.

The stable isotope analyses of the femoral bone sample showed a δ^13^C value of -19.6‰ that indicates a high proportion of C_3_-plants such as cereals in the diet. Similar values could be observed for historical individuals from present-day Switzerland and Liechtenstein. A male traveller dating to approximately 1600 AD found at the Theodul glacier and died while crossing the alps between Switzerland and Italy shows a similar value of -19.9‰ [64]. An Iron Age population (n = 63) from Münsingen near Bern is with a mean value of -19.5‰ in the same range [34]. Moreover, two adult males from Bronze Age Liechtenstein show very comparable δ^13^C data of -20.3‰ [65]. Hence, Jenatsch’s value fits to the other values from regional archaeological human remains. In comparison to them his value is slightly more positive and therefore might reflect a diet that included a certain amount of C_4_-plants such as millet. In fact, a signal for millet has been inferred from δ^13^C values spanning the Bronze Age to the Middle Ages of northern Italy and the Balkan [66,67], and millet grains have been found in rubbish pits in medieval Zürich and southern Germany [68,69]. In the Middle Ages, millet was mainly consumed in the form of porridge and soup and is considered the staple food of the poor [70].

Due to the relatively slow turnover rate of collagen from the femoral cortex, the δ^13^C values reflect an individual’s diet over at least 10 years prior to death, but it may include a substantial portion of older collagen [28]. Hence, this signal for millet may well point to Jörg Jenatsch’s adolescence. However, the lack of reference data for individuals from the 17^th^ century of Graubünden limits the interpretation of the δ^13^C data.

Jenatsch’s δ^15^N value of 10.3‰ is remarkably high in comparison to the other data from the area of Switzerland [34,65,71,72]. It is clearly above the herbivore baseline of 2–7‰ [72,73] and thus indicates a high intake of animal protein such as meat and dairy products. Even though meat consumption was not restricted to people of higher social classes, they ate proportionally more meat relative to other food compared to poorer people [70]. The δ^15^N value might, however, also result from fish consumption [67] and thus could reflect the various journeys Jenatsch made to Venice, Friuli and France [2]. In 1629, he was also imprisoned in Venice for several months [2], where sea fish was probably a significant food component. However, salted herrings were also very common in Switzerland for the better-off people since the High Middle Ages [74].

A diet slightly enriched by seafood would be in agreement with Jörg Jenatsch’s stable sulphur isotope value of 6.2‰. While organisms in marine ecosystems have δ^34^S values around +20‰, terrestrial mammals have values lower than +10‰ [75]. Jenatsch’s value is, however, higher than the mean of the published δ^34^S data from Switzerland and Liechtenstein, which are 1.0‰ and 1.8‰ [34,65]. However, the value is similar to that of the 1600 AD traveller (6.1‰) found at the Theodul glacier [64].

The strontium and lead isotope ratios of Jörg Jenatsch show highly significant differences between tooth enamel and dentin. This indicates a significant mobility during his life [76]. Even though it cannot be excluded that porous dentine exchanges Sr with the surrounding burial sand [77], this would fit his biographical data, as he spent his childhood mostly in the upper Engadin, a high valley in southern Graubünden, studied in Zurich and Basel, became pastor in the Domleschg in middle Graubünden and then in the Valtellina, which at that time belonged to Graubünden and is geologically similar to the nearby Engadin, before he travelled much as mercenary under the French-Venice and the Austrian-Habsburg crowns, respectively.

In sum, our recent comprehensive analysis of the remains dispels any initial doubts about the identity of this skeleton found in 1959 close to the presumed original location of Jörg Jenatsch’s gravestone in the Chur cathedral. Our data confirm that the skeleton most likely belongs to this controversial freedom fighter of the Thirty Year’s War, Jörg Jenatsch.

## References

[1] Haffter E (1894) Georg Jenatsch—ein Beitrag zur Geschichte der Bündner Wirren. Davos: Richter 552 p.

[2] Pfister A (1991) Jörg Jenatsch—sein Leben und seine Zeit. 5th edn (with a chapter by Jon Mathieu; 1st edn appeared 1938 with the title "Georg Jenatsch—Sein Leben und seine Zeit. Zu seinem dreihundertsten Todestage"). Chur: Verlag Bündner Monatsblatt.

[3] Head RC (2008) Jenatsch's Axe—Social Boundaries, Identity, and Myth in the Era of the Thirty Years' War. Rochester, N.Y.: University of Rochester Press.

[4] Janosa M, Archäologischer Dienst Graubünden, editors (2014) unter die orgl begraben—das Grab des Jörg Jenatsch in der Kathedrale zu Chur. Chur: Somedia-Buchverlag.

[5] Meyer CF (1876) Jürg Jenatsch—eine Bündnergeschichte. Leipzig: Haessel.

[6] Schmid D (1987) Jenatsch (DVD). Zürich: Limbo Film.

[7] Berger M (1960) Wer hat Jenatsch ermordet? Bündner Monatsblatt 1960: 153–192.

[8] Jecklin F, Valèr M (1924) Die Ermordung Georg Jenatschs. Nach dem Churer Verhörprotokoll; kommentiert von M. Valèr. Zeitschrift für Schweizerische Geschichte 4: 396–444.

[9] Pfister A, editor (1983) Jörg Jenatsch—Briefe 1614–1639. Chur: Terra Grischuna.

[10] Janosa M (2010) Die Exhumierung des Jörg Jenatsch im Jahre 1959. Bündner Monatsblatt 2010 (5): 431–452.

[11] Hug E (1911–1991) Unpublished documents concerning the discovery of the grave of Jörg Jenatsch in the Chur cathedral, inv. No. A Sp III/15q. Chur: Staatsarchiv Graubünden.

[12] Fischer E, Saller K (1928) Eine neue Haartafel. Anthropol Anz 5: 238–244; 288.

[13] Martin R, Saller K (1957) Lehrbuch der Anthropologie. Stuttgart: Fischer.

[14] Haas C, Shved N, Rühli FJ, Papageorgopoulou C, Purps J, Geppert M, et al (2013) Y-chromosomal analysis identifies the skeletal remains of Swiss national hero Jörg Jenatsch (1596–1639). Forensic Sci Int Genet 7: 610–617. 24035510 10.1016/j.fsigen.2013.08.006

[15] Walsh S, Liu F, Wollstein A, Kovatsi L, Ralf A, Kosiniak-Kamysz A, et al (2013) The HIrisPlex system for simultaneous prediction of hair and eye colour from DNA. Forensic Sci Int Genet 7: 98–115. 22917817 10.1016/j.fsigen.2012.07.005

[16] Acsádi G, Nemeskéry J (1970) History of Human Life Span and Mortality. Budapest: Akadémiai Kiadó 346 p.

[17] Rösing FW, Graw M, Marré B, Ritz-Timme S, Rothschild MA, Rötzscher K, et al (2007) Recommendations for the forensic diagnosis of sex and age from skeletons. Homo 58: 75–89. 10.1016/j.jchb.2005.07.002 17306261

[18] Wilkinson C (2004) Forensic Facial Reconstruction. Cambridge: Cambridge University Press 290 p.

[19] Helmer R (1984) Schädelidentifizierung durch elektronische Bildmischung. Zugleich ein Beitrag zur Konstitutionsbiometrie und Dickenmessung der Gesichtsweichteile. Heidelberg: Kriminalistik-Verlag.

[20] Prag J, Neave R (1997) Making Faces: Using Forensic and Archaeological Evidence. London: British Museum.

[21] Cooper A, Poinar HN (2000) Ancient DNA: do it right or not at all. Science 289: 1139 10970224 10.1126/science.289.5482.1139b

[22] Gilbert MT, Bandelt HJ, Hofreiter M, Barnes I (2005) Assessing ancient DNA studies. Trends Ecol Evol 20: 541–544. 10.1016/j.tree.2005.07.005 16701432

[23] Morin PA, McCarthy M (2007) Highly accurate SNP genotyping from historical and low-quality samples. Mol Ecol Notes 7: 937–946.

[24] Taberlet P, Griffin S, Goossens B, Questiau S, Manceau V, Escaravage N, et al (1996) Reliable genotyping of samples with very low DNA quantities using PCR. Nucleic acids research 24: 3189–3194. 8774899 10.1093/nar/24.16.3189 PMC146079

[25] Ambrose SH (1990) Preparation and characterization of bone and tooth collagen for isotopic analysis. J Archaeol Sci 17: 431–451.

[26] Longin R (1971) New Method of Collagen Extraction for Radiocarbon Dating. Nature 230: 241–242. 4926713 10.1038/230241a0

[27] Hoefs J (2009) Stable Isotope Geochemistry. Berlin: Springer.

[28] Hedges REM, Clement JG, Thomas CDL, O'Connell TC (2007) Collagen turnover in the adult femoral mid-shaft: modeled from anthropogenic radiocarbon tracer measurements. Am J Phys Anthropol 133: 808–816. 10.1002/ajpa.20598 17405135

[29] DeNiro MJ, Epstein S (1978) Influence of diet on the distribution of carbon isotopes in animals. Geochim Cosmochim Ac 42: 495–506.

[30] Bocherens H, Drucker D (2003) Trophic level isotopic enrichment of carbon and nitrogen in bone collagen: Case studies from recent and ancient terrestrial ecosystems. Int J Osteoarchaeol 13: 46–53.

[31] Ambrose SH (1993) Isotopic analysis of paleodiets: Methodological and interpretive considerations In: Sanford MK, editor. Investigations of Ancient Human Tissue—Chemical Analyses in Anthropology. Amsterdam: Gordon and Breach pp. 59–130.

[32] Lösch S, Grupe G, Peters J (2006) Stable isotopes and dietary adaptations in humans and animals at Pre-Pottery Neolithic Nevali Cori, southeast Anatolia. Am J Phys Anthropol 131: 181–193. 10.1002/ajpa.20395 16596597

[33] Lösch S, Moghaddam N, Grossschmidt K, Risser DU, Kanz F (2014) Stable isotope and trace element studies on gladiators and contemporary romans from Ephesus (Turkey, 2nd and 3rd c AD)—Implications for differences in diet. Plos One 9: e110489 10.1371/journal.pone.0110489 25333366 PMC4198250

[34] Moghaddam N, Müller F, Hafner A, Lösch S (2016) Social stratigraphy in Late Iron Age Switzerland: stable carbon, nitrogen and sulphur isotope analysis of human remains from Münsingen. Archaeol Anthropol Sci: 8: 149–160.

[35] Vika E (2009) Strangers in the grave? Investigating local provenance in a Greek Bronze Age mass burial using δ^34^S analysis. J Archaeol Sci 36: 2024–2028.

[36] Grupe G, Price TD, Schröter P, Söllner F, Johnson CM, Beard BL (1997) Mobility of Bell Beaker people revealed by strontium isotope ratios of tooth and bone: a study of southern Bavarian skeletal remains. Appl Geochem 12: 517–525.

[37] Hoppe KA, Koch PL, Furutani TT (2003) Assessing the preservation of biogenic strontium in fossil bones and tooth enamel. Int J Osteoarchaeol 13: 20–28.

[38] Åberg G, Fosse G, Stray H (1998) Man, nutrition and mobility: a comparison of teeth and bone from the medieval era and the present from Pb and Sr isotopes. Sci Total Environ 224: 109–119. 9926429 10.1016/s0048-9697(98)00347-7

[39] Montgomery J, Evans JA, Powlesland D, Roberts CA (2005) Continuity or colonization in Anglo-Saxon England? Isotope evidence for mobility, subsistence practice, and status at West Heslerton. Am J Phys Anthropol 126: 123–138. 10.1002/ajpa.20111 15386290

[40] Villa IM (2009) Lead isotopic measurements in archeological objects. Archaeol Anthropol Sci 1: 149–153.

[41] Villa IM, Ruggieri G, Puxeddu M, Bertini G (2006) Geochronology and isotope transport systematics in a subsurface granite from the Larderello-Travale geothermal system (Italy). J Volcanol Geotherm Res 152: 20–50.

[42] Herrmann B, Grupe G, Hummel S, Piepenbrink H, Schutkowski H (1990) Prähistorische Anthropologie: Leitfaden der Feld- und Labormethoden. Berlin: Springer IX, 445 p.

[43] Ruff CB, Holt BM, Niskanen M, Sladék V, Berner M, Garofalo E, et al (2012) Stature and body mass estimation from skeletal remains in the European Holocene. Am J Phys Anthropol 148: 601–617. 10.1002/ajpa.22087 22639191

[44] Lovejoy CO (1985) Dental wear in the Libben population: its functional pattern and role in the determination of adult skeletal age at death. Am J Phys Anthropol 68: 47–56. 10.1002/ajpa.1330680105 4061601

[45] Prokop O, Göhler W, Bernt A (1976) Forensische Medizin. Stuttgart: Fischer.

[46] Walsh S, Wollstein A, Liu F, Chakravarthy U, Rahu M, Seland JH, et al (2012) DNA-based eye colour prediction across Europe with the IrisPlex system. Forensic Sci Int Genet 6: 330–340. 10.1016/j.fsigen.2011.07.009 21813346

[47] DeNiro MJ (1985) Postmortem preservation and alteration of invivo bone-collagen isotope ratios in relation to paleodietary reconstruction. Nature 317: 806–809.

[48] Haffter E (1895) Georg Jenatsch—Urkundenbuch enthaltend Exkurse und Beilagen. Chur: Hitz'sche Buchhandlung.

[49] Steele J (2000) Handedness in past human populations: Skeletal markers. Laterality: Asymmetries of Body, Brain and Cognition 5: 193–220. 10.1080/713754380 15513142

[50] Lean MEJ, Han TS, Morrison CE (1995) Waist circumference as a measure for indicating need for weight management. BMJ 311: 158–161. 7613427 10.1136/bmj.311.6998.158 PMC2550221

[51] Austin-Smith D, Maples WR (1994) The reliability of skull photograph superimposition in individual identification. J Forensic Sci 39: 446–455. 8195756

[52] Damas S, Cordón O, Ibáñez O, Santamaría J, Alemán I, Botella M, et al (2011) Forensic identification by computer-aided craniofacial superimposition: a survey. ACM Comput Surv 43: 27/21–27.

[53] Jayaprakash PT, Srinivasan GJ, Amravaneswaran MG (2001) Cranio-facial morphanalysis: a new method for enhancing reliability while identifying skulls by photo superimposition. Forensic Sci Int 117: 121–143. 11230953 10.1016/s0379-0738(00)00455-2

[54] Walsh S, Liu F, Ballantyne KN, van Oven M, Lao O, Kayser M (2011) IrisPlex: a sensitive DNA tool for accurate prediction of blue and brown eye colour in the absence of ancestry information. Forensic Sci Int Genet 5: 170–180. 10.1016/j.fsigen.2010.02.004 20457092

[55] Brothwell DR, Spearman R (1963) The hair of earlier peoples In: Brothwell D, Higgs E, editors. Science in Archaeology A Comprehensive Survey of Progress and Research. London: Thames and Hudson pp. 427–436.

[56] Appleby J, Rutty GN, Hainsworth SV, Woosnam-Savage RC, Morgan B, Brough A, et al (2015) Perimortem trauma in King Richard III: a skeletal analysis. The Lancet 385: 253–259. 10.1016/S0140-6736(14)60804-7 25238931

[57] Buckley R, Morris M, Appleby J, King T, O'Sullivan D, Foxhall L (2013) ‘The king in the car park’: new light on the death and burial of Richard III in the Grey Friars church, Leicester, in 1485. Antiquity 87: 519–538.

[58] Lamb AL, Evans JE, Buckley R, Appleby J (2014) Multi-isotope analysis demonstrates significant lifestyle changes in King Richard III. J Archaeol Sci 50: 559–565.

[59] King TE, Fortes GG, Balaresque P, Thomas MG, Balding D, Delser PM, et al (2014) Identification of the remains of King Richard III. Nat Commun 5: 6631. 10.1038/ncomms6631 PMC4268703 25463651

[60] Papageorgopoulou C (2008) The medieval population of Tomils (11th– 15th c AD): an archaeo-anthropological approach. PhD Thesis, Basel: Universität Basel.

[61] Koepke N, Baten J (2005) The biological standard of living in Europe during the last two millennia. European Review of Economic History 9: 61–95.

[62] Papageorgopoulou C, Suter SK, Rühli FJ, Siegmund F (2011) Harris lines revisited: prevalence, comorbidities, and possible etiologies. Am J Hum Biol 23: 381–391. 10.1002/ajhb.21155 21387459

[63] Lopez B, Pardiñas AF, Garcia-Vazquez E, Dopico E (2012) Socio-cultural factors in dental diseases in the Medieval and early Modern Age of northern Spain. Homo 63: 21–42. 10.1016/j.jchb.2011.12.001 22265008

[64] Alterauge A, Providoli S, Moghaddam N, Lösch S (2015) Death in the ice—Re-investigations of the remains from the Theodul glacier (Switzerland). Journal of Glacial Archaeology 2: 35–50.

[65] Cooper C, Lösch S, Mayr U, Moghaddam N, Stehrenberger T (2011) Triesen, Fürst-Johann-Strasse 40 In: Frommelt H, Stehrenberger T, editors. Denkmalpflege und Archäologie im Fürstentum Liechtenstein—Fund und Forschungsberichte 2011. Vaduz: Hochbauamt des Fürstentums Liechtenstein, Denkmalpflege und Archäologie pp. 136–153.

[66] Iacumin P, Galli E, Cavalli F, Cecere L (2014) C4-consumers in southern Europe: The case of Friuli V.G. (NE-Italy) during Early and Central Middle Ages. Am J Phys Anthropol 154: 561–574. 10.1002/ajpa.22553 24889200

[67] Reitsema LJ, Vercellotti G (2012) Stable isotope evidence for sex- and status-based variations in diet and life history at medieval Trino Vercellese, Italy. Am J Phys Anthropol 148: 589–600. 10.1002/ajpa.22085 22553011

[68] Küster H (1992) Pflanzliche Ernährung In: Flüeler M, Flüeler N, editors. Stadtluft, Hirsebrei und Bettelmönch—Die Stadt um 1300. Stuttgart: Theiss pp. 289–293.

[69] Meyer W (1985) Hirsebrei und Hellebarde—auf den Spuren des mittelalterlichen Lebens in der Schweiz. Olten: Walter.

[70] Adamson MW (2004) Food in Medieval Times. Westport: Greenwood Press.

[71] Alterauge A, Providoli S, Moghaddam N, Lösch S (2015) Death in the ice—Re-investigations of the remains from the Theodul glacier (Switzerland). Journal of Glacial Archaeology 2: 35–50.

[72] Tütken T, Langenegger E, Wild W (2008) Einheimisch oder fremd? Isotopenanalyse eines Frauenskelettes des 9. Jahrhunderts n. Chr. aus Elsau, Kanton Zürich, Schweiz. Anthropol Anz 66: 19–50. 18435204

[73] Müldner G, Richards MP (2007) Stable isotope evidence for 1500 years of human diet at the city of York, UK. Am J Phys Anthropol 133: 682–697. 10.1002/ajpa.20561 17295296

[74] Amacher U (2006) Geschichte der Fischer und der Fischerei im Mittelalter In: Hüster-Plogmann H, editor. Fisch und Fischer aus zwei Jahrtausenden Eine fischereiwirtschaftliche Zeitreise durch die Nordwestschweiz. Augst: Römerstadt Augusta Raurica pp. 95–106.

[75] Richards MP, Fuller BT, Hedges REM (2001) Sulphur isotopic variation in ancient bone collagen from Europe: implications for human palaeodiet, residence, mobility, and modern pollutant studies. Earth and Planetary Sciences 191: 185–190.

[76] Price TD, Burton JH, Bentley RA (2002) The characterization of biologically available strontium isotope ratios for the study of prehistoric migration. Archaeometry 44: 117–135.

[77] Bentley RA (2006) Strontium isotopes from the earth to the archaeological skeleton: A review. Journal of Archaeological Method and Theory 13: 135–187.

